# Potential dopaminergic deficit in patients with geriatric psychiatric disorders as revealed by DAT-SPECT: a cross-sectional study

**DOI:** 10.1136/bmjment-2024-301042

**Published:** 2024-07-30

**Authors:** Shintaro Takenoshita, Seishi Terada, Katsuhide Kojima, Naoto Nishikawa, Tomoko Miki, Osamu Yokota, Masaki Fujiwara, Manabu Takaki

**Affiliations:** 1 Department of Neuropsychiatry, Okayama University Hospital, Okayama, Japan; 2 Department of Neuropsychiatry, Okayama University Faculty of Medicine Dentistry and Pharmaceutical Sciences, Okayama, Japan; 3 Department of Radiology, Okayama University Hospital, Okayama, Japan; 4 Department of Psychiatry, Kinoko Espoir Hospital, Kasaoka, Okayama, Japan

**Keywords:** Depression & mood disorders, Delirium & cognitive disorders, Cross-Sectional Studies

## Abstract

**Background:**

It has been reported that patients with geriatric psychiatric disorders include many cases of the prodromal stages of neurodegenerative diseases. Abnormal ^123^I-2β-carbomethoxy-3β-(4-iodophenyl)-N-(3-fluoropropyl) nortropane dopamine transporter single-photon emission computed tomography (DAT-SPECT) reveals a nigrostriatal dopaminergic deficit and is considered useful to detect dementia with Lewy bodies and Parkinson’s disease as well as progressive supranuclear palsy and corticobasal degeneration. We aimed to determine the proportion of cases that are abnormal on DAT-SPECT in patients with geriatric psychiatric disorders and to identify their clinical profile.

**Methods:**

The design is a cross-sectional study. Clinical findings of 61 inpatients aged 60 years or older who underwent DAT-SPECT and had been diagnosed with psychiatric disorders, but not neurodegenerative disease or dementia were analysed.

**Results:**

36 of 61 (59%) had abnormal results on DAT-SPECT. 54 of 61 patients who had DAT-SPECT (89%) had undergone ^123^I-metaiodobenzylguanidine myocardial scintigraphy (^123^I-MIBG scintigraphy); 12 of the 54 patients (22.2%) had abnormal findings on ^123^I-MIBG scintigraphy. There were no cases that were normal on DAT-SPECT and abnormal on ^123^I-MIBG scintigraphy. DAT-SPECT abnormalities were more frequent in patients with late-onset (55 years and older) psychiatric disorders (69.0%) and depressive disorder (75.7%), especially late-onset depressive disorder (79.3%).

**Conclusion:**

Patients with geriatric psychiatric disorders include many cases showing abnormalities on DAT-SPECT. It is suggested that these cases are at high risk of developing neurodegenerative diseases characterised by a dopaminergic deficit. It is possible that patients with geriatric psychiatric disorders with abnormal findings on DAT-SPECT tend to show abnormalities on DAT-SPECT first rather than on ^123^I-MIBG scintigraphy.

**WHAT IS ALREADY KNOWN ON THIS TOPIC**

- It has been reported that patients with geriatric psychiatric disorders include cases in the prodromal stages of neurodegenerative diseases. However, the proportion of geriatric psychiatric disorders with a background of neurodegenerative disorders and the pattern of findings of imaging biomarkers of these cases have not been identified.

**WHAT THIS STUDY ADDS**

- This study revealed that many patients with geriatric psychiatric disorders show abnormal findings on dopamine transporter single-photon emission computed tomography (DAT-SPECT), especially patients with late-onset psychiatric disorders and depressive disorders. It was also found that patients with geriatric psychiatric disorders with abnormal DAT-SPECT results tend to show abnormalities on DAT-SPECT, but not on ^123^I-metaiodobenzylguanidine myocardial scintigraphy.

**HOW THIS STUDY MIGHT AFFECT RESEARCH, PRACTICE OR POLICY**

- Clinicians need to recognise that patients with geriatric psychiatric disorders have a high risk of developing neurodegenerative diseases characterised by a dopaminergic deficit. Further studies should examine more appropriate treatment approaches for this group.

## Introduction

 Among neurodegenerative diseases, there are cases in which the phenotype in the prodromal phase of the disease is not cognitive or motor impairment, but psychiatric disturbance. For instance, in Lewy body disease (LBD), the clinical phenotype of dementia with Lewy bodies (DLB) in the prodromal stage is known to have several subtypes, including psychiatric-onset DLB,^1 2^ which is manifested by depression and psychosis. Parkinson’s disease (PD) has also been reported to present as depression and anxiety in the prodromal stage.^3 4^ Cases of neurodegenerative diseases that develop as psychiatric disorders have received much attention not only in LBD but also in progressive supranuclear palsy (PSP) and corticobasal degeneration (CBD).^5 6^

Differentiating psychiatric disorders with and without a background of neurodegenerative diseases allow for more appropriate treatment options. For example, patients with DLB are characterised as responding well to cholinesterase inhibitors, being very sensitive to neuroleptics and developing severe extrapyramidal side effects.^7^ Early detection of cases of psychiatric disturbance with a background of neurodegenerative diseases allows for effective treatment options while avoiding adverse events.

Recent studies have reported that attempted to detect psychiatric disturbance with a background of neurodegenerative diseases using various imaging modalities. Abnormal ^123^I-2β-carbomethoxy-3β-(4-iodophenyl)-N-(3-fluoropropyl) nortropane (^123^I-FP-CIT) dopamine transporter single-photon emission computed tomography (DAT-SPECT) suggests a nigrostriatal dopaminergic deficit and is considered useful to detect LBD such as DLB and PD, as well as multiple system atrophy (MSA), PSP and CBD.^8^,^10^ Reduced ^123^I-metaiodobenzylguanidine myocardial scintigraphy (^123^I-MIBG scintigraphy) uptake is considered a biomarker indicative of LBD.^11 12^ With regard to patients with geriatric psychiatric disorders, one study reported that DAT-SPECT performed on patients with depressive disorder diagnosed after the age of 55 years or older and found abnormalities in 24% (7/29) of them,^13^ and another study reported that ^123^I-MIBG scintigraphy found abnormalities in 27% (14/52) of depressed patients over 60 years old.^14^ These results suggest that it is not uncommon for patients in the prodromal phase of a neurodegenerative disease such as DLB to visit psychiatrists and be diagnosed with geriatric depression.

Other imaging modalities may also be useful as a means of distinguishing neurodegenerative diseases from geriatric psychiatric disorders. Assessment of atrophy using structural imaging by MRI is a useful method to discriminate PSP from other neurodegenerative diseases using measurement of the midbrain area on midsagittal MRI has been reported as a method that can differentiate PSP from PD, MSA and normal ageing.^15^ Decreased blood flow in the occipital lobe and relative retention in the posterior cingulate gyrus revealed by cerebral blood flow scans/metabolism scans of the so-called the cingulate island sign (CIS) are reported to be useful to distinguish between DLB and Alzheimer’s disease of the mild cognitive impairment type.^16^

However, no study has clarified the patterns of findings on DAT-SPECT and other imaging biomarkers such as ^123^I-MIBG scintigraphy in patients with geriatric psychiatric disorders. The purpose of this study was to determine the pattern of findings on imaging biomarkers in patients with geriatric psychiatric disorders and to identify the clinical profile of cases with abnormalities on DAT-SPECT.

## Methods

### Subjects

Between January 2015 and January 2021, we recruited participants from inpatients with geriatric psychiatric disorders in the Department of Neuropsychiatry ward of Okayama University Hospital. Our inclusion criteria were: (1) age of 60 years and older and (2) having been given a definitive diagnosis by the chief psychiatrist on admission according to The Diagnostic and Statistical Manual of Mental Disorders, Fifth Edition.^17^ Exclusion criteria were: (1) diagnosis of dementia, neurodegenerative disease or delirium, (2) admission for examination or rest only, not treatment or (3) admission for the treatment of a corporal illness such as cancer. For patients who were hospitalised more than once during the study period, data from only the first hospitalisation were included in the study.

### Evaluation of clinical information

The imaging findings of each patient were excerpted from inpatient charts by a research investigator who was different from the physician in charge of the patient’s treatment. Information such as age, age of onset, gender, years of education, psychiatric diagnosis, results of various neuropsychological tests, parkinsonism, hallucinations, cognitive fluctuations, REM sleep behaviour disorder (RBD), other psychiatric symptoms and physical symptoms were evaluated using inpatient charts and nursing records by a research investigator who was blind to the results of any imaging examination. For the purposes of this study, cases diagnosed at the age of 55 years and older were defined as late-onset psychiatric disorders, while cases diagnosed before the age of 55 years were defined as early-onset psychiatric disorders with reference to previous research.^13^ All patients were assessed using the Hamilton Depression Rating Scale (HDRS) at the time of admission.^18^ The attending physician performed a cognitive function test on all patients. With the exception of those patients who refused testing and those whom the physician deemed that testing was inappropriate, the cognitive abilities of participants were evaluated by the following neuropsychological tests: the Mini-Mental State Examination (MMSE),^19 20^ Frontal Assessment Battery (FAB)^21 22^ and the Clock Drawing Test (CDT).^23^ Olfactory function was assessed by the odour stick identification test for Japanese (OSIT-J).^24^

### DAT-SPECT imaging

Four hours after slow injection of 167 MBq of ^123^I-FP-CIT (DaT SCAN; Nihon Medi-Physics, Tokyo, Japan), projection data were obtained using two SPECT systems: a dual-head hybrid SPECT/CT system (Symbia T16; Siemens Healthcare, Erlangen, Germany) using a fan-beam collimator reconstructed by Flash 3D (Siemens Healthcare) and a triple-head SPECT system (GCA9300R; Canon Medical Systems, Tochigi, Japan) using the fan-beam high-resolution collimator reconstructed by the three-dimensional ordered subset expectation maximisation (3D-OSEM) method (iteration 10, subset 10). Differences in specific binding ratio (SBR) values between the two systems were corrected using a striatal phantom. When reconstructed with attenuation correction and scatter correction, the matrix size was 128 × 128. The image acquisition time was 28 min. The SBR was calculated as the semi-quantitative evaluation. This method consisted of collecting all the radioactivity from the striatum of each hemisphere and estimating the background radioactivity from the whole brain minus that from the striatum. The SBR was semi-quantitatively calculated using DAT VIEW software (Nihon Medi-Physics, Tokyo, Japan) based on Bolt’s method as described in detail elsewhere.^25^ A database from a previous study generated using ^123^I-FP-CIT SPECT data from 256 healthy Japanese subjects (age range 30–83 years) was used as the healthy control group.^26^ In this study, the SBR was considered abnormal when either the left or right SBR was less than −2 SD of the mean value calculated from the value of the control group.

### ^123^I-MIBG myocardial scintigraphy imaging

Cardiac planar images were acquired 15 min (early image) and 4 hours (late image) after the injection of 111 MBq of ^123^I-MIBG (PDRadiopharma, Tokyo, Japan) using a dual-head hybrid SPECT/CT system (Discovery NM/CT670 Pro, GE Healthcare, Chicago, Illinois, USA) equipped with an extended low-energy general-purpose (ELEGP) collimator. The image acquisition time was 5 min. The cardiac uptake of MIBG was determined by setting a region of interest (ROI) in the left cardiac ventricle and upper mediastinum using a semiautomatic ROI setting software programme.^21^ The average counts per pixel in the heart (H) and mediastinum (M) were determined within each ROI to calculate the H/M ratio at 15 min (early image) and 4 hours (late image). Because the cut-off value for the H/M ratios widely used in studies is 2.2, the group with a heart-to-mediastinum (H/M) ratio<2.2 in the late image was defined as the low uptake group in this study.^12^

### Head MRI imaging

T1-weighted image (T1WI), T2-weighted image (T2WI) and fluid-attenuated inversion recovery (FLAIR) image were obtained with 3T scanners (MAGNETOM Prisma, MAGNETOM Skyra and MAGNETOM Verio; Siemens Healthcare). White matter changes were defined as ill-defined hyperintensities on T2WI and FLAIR images without any abnormality on T1WI, and as ill-defined and moderately hypodense areas on CT. White matter changes were graded from 0 to 3 in the right and left hemispheres separately for each brain region (frontal, parieto-occipital, temporal, basal ganglia, infratentorial), using the Age-Related White Matter Change (ARWMC) Rating Scale.^27^ All patients were offered MRI preferentially, but CT was performed as a second option for those cases in which the attending physician deemed MRI inappropriate. The area of the midbrain tegmentum was measured on midsagittal T1WI of MRI using the display tools of a workstation. The area of the midbrain could not be measured in patients for whom only CT was taken. In this study, patients with midbrain area measurement of <100.0 mm^2^ were defined as the small group referring to a previous study.^15^

### Brain perfusion SPECT

Imaging began 10 min after intravenous administration of technetium-99m ethyl cysteinate dimer (ECD) 740 MBq (PDRadiopharma). SPECT images were obtained with two SPECT systems: a dual-head hybrid SPECT/CT system (Symbia T16; Siemens Healthcare) using a fan-beam collimator reconstructed by Flash 3D (Siemens Healthcare) and a triple-head SPECT system (GCA9300R; Canon Medical Systems) using the fan-beam high-resolution collimator reconstructed by the 3D-OSEM method (iteration 10, subset 10). The reconstructed matrix size with attenuation correction and scatter correction was 128 × 128. The easy Z-score imaging system (eZIS) was used to evaluate the CIS score by regional cerebral blood flow (rCBF). A detailed description of eZIS has been included in previous studies.^28^ In summary, SPECT images of each patient were subjected to standardisation corrections and then compared with a control database obtained from healthy controls in each age group, from which volume of interest (VOI) scores were calculated by automated analysis. The CIS score is obtained by dividing the total Z-score on the reduced rCBF in the VOI-2 (occipital cingulate gyrus), defined as the region in which rCBF is significantly reduced in DLB patients compared with healthy controls, by the total Z-score on the reduced rCBF in VOI-1 (posterior cingulate gyrus), defined as the region in which rCBF is significantly reduced in patients with Alzheimer’s disease (AD).^28^ A CIS core of less than 0.281 can distinguish DLB from AD with a sensitivity of 92.3% and specificity of 76.9%.^28^ In this study, a CIS score<0.281 was considered CIS positive.

### Statistical analysis

Statistical analysis was performed using the SPSS V.24.0 software programme (IBM, Armonk, New Y0rk, USA). Comparisons of continuous variables between the two independent groups were performed by independent sample t-tests for normally distributed data or the Mann-Whitney U-test for non-normally distributed data. Comparison between the proportions of two independent groups was calculated using Fisher’s exact test (for smaller numbers) or the χ^2^ test. A value of p<0.05 was accepted as significant.

## Results

**Figure 1:**
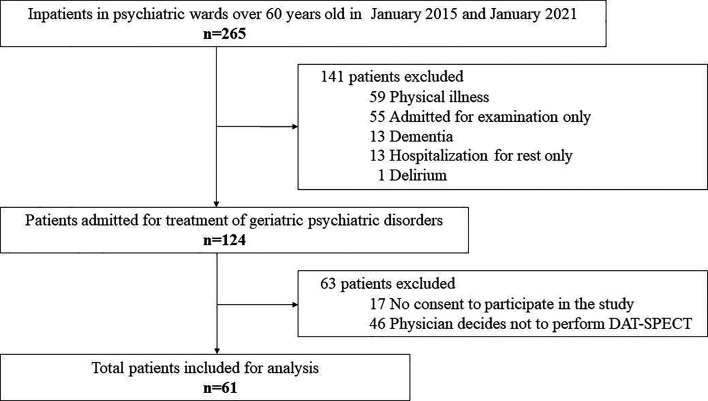
Flow diagram of the study. DAT-SPECT, dopamine transporter single-photon emission computed tomography.

Of the 265 inpatients with geriatric psychiatric disorders (≥60 years old), 141 were excluded for meeting the exclusion criteria and 124 were eligible (figure 1). Of the 124 eligible patients, 61 were finally included, excluding patients from whom consent to participate in the study could not be obtained and patients for whom the clinical physician deemed it problematic to perform examinations due to concerns about the physical or psychological invasion that DAT-SPECT would cause. DAT-SPECT was not performed in some cases due to a lack of patient consent or its invasiveness, but in no case was the test not performed based on the clinical judgement of the attending physician alone.

### Psychiatric diagnoses

The psychiatric diagnoses of the 61 patients included 37 with major depressive disorder (MDD), 9 with schizophrenia, 7 with bipolar disorder, 4 with delusional disorder, and 4 with anxiety disorder. Details of the psychiatric diagnoses of potential participants, included patients and excluded patients are shown in online supplemental table e-1.

### Dopamine transporter single-photon emission computed tomography

Characteristics of the patients who underwent DAT-SPECT are shown in table 1. Of the 61 patients, 36 (59.0%) were abnormal on DAT-SPECT. There were no differences between abnormal and normal DAT-SPECT groups with respect to current age, gender, years of education and scores of neuropsychological tests. Depression was significantly more common in the abnormal DAT-SPECT group than in the normal group. There were no differences in clinical symptoms between the abnormal and normal DAT-SPECT groups (table 1 and online supplemental table e-2). There were significantly more patients taking SSRI (selective serotonin reuptake inhibitors) and SNRI (selective serotonin–norepinephrine reuptake inhibitors) and significantly fewer patients taking benzodiazepine in the abnormal DAT-SPECT group than in the normal group (table 1). There were no significant differences between the abnormal and normal DAT-SPECT groups in patients taking other psychotropics and other medications. No patient was taking psychostimulants.

**Table 1:** Characteristics of the patients who underwent DAT-SPECT

	Abnormal DAT-SPECT (n=36)	Normal DAT-SPECT (n=25)	*t*	P value
Sex, n (female/male)	27/9	16/9		0.354
Age in years, mean (SD)	72.7 (6.2)	71.4 (7.3)	0.732	0.467
Age at onset in years, mean (SD)	62.6 (12.0)	55.6 (15.8)	1.954	0.055
Late onset/early onset (%)	29/7 (80.6)	13/12 (52.0)		0.018*
Education in years, mean (SD)	13.0 (2.8)	11.4 (2.7)	1.925	0.061
^123^I-MIBG, abnormal/normal (%)	12/20 (37.5)	0/22 (0)		0.001*
CIS, ± (%)	16/17 (47.1)	15/10 (60.0)		0.384
ARWMC score, more than 5/less than 5 (%)	20/16 (55.6)	7/18 (28.0)		0.033*
ARWMC score of basal ganglia, 1 and above/0 (%)	13/23 (36.1)	0/25 (0)		<0.001*
Midbrain atrophy, ± (%)	5/30 (14.3)	2/23 (8.0)		0.455
Neuropsychological tests†				
HDRS, mean (SD)	19.6 (7.7)	18.0 (9.4)	0.547	0.587
MMSE, mean (SD)	25.8 (3.8)	26.3 (3.0)	−0.440	0.662
FAB, mean (SD)	12.6 (1.5)	14.1 (2.8)	−1.488	0.147
CDT, mean (SD)	8.9 (1.4)	8.9 (1.5)	0.076	0.940
OSIT-J, mean (SD)	4.3 (3.2)	5.1 (3.1)	−0.400	0.694
Clinical symptoms				
Depression, n (%)	31 (86.1)	15 (60.0)		0.020*
Anxiety, n (%)	22 (61.1)	19 (76.0)		0.223
Delusion, n (%)	15 (41.7)	16 (64.0)		0.086
Fluctuations, n (%)	5 (13.9)	5 (20.0)		0.727
Parkinsonism, n (%)§	13 (36.1)	6 (24.0)		0.315
Visual hallucination, n (%)	2 (5.6)	2 (8.0)		1.000
REM sleep behaviour disorder, n (%)	2 (5.6)	0 (0)		0.508
Medications‡				
SSRI, n (%)	16 (44.4)	3 (12.0)		0.007*
SNRI, n (%)	7 (19.4)	0 (0)		0.019*
NaSSA, n (%)	8 (22.2)	5 (20.0)		1.000
Tricyclic, n (%)	1 (2.8)	0 (0)		0.500
Tetracyclic, n (%)	0 (0)	0 (0)		–
SARI, n (%)	8 (22.2)	3 (12.0)		0.500
Benzodiazepine, n (%)	18 (50.0)	20 (80.0)		0.017*
Mood stabiliser, n (%)	6 (16.7)	4 (16.0)		1.000
Antipsychotic drug, n4242 (%)	17 (47.2)	14 (56.0)		0.500
Calcium-blocker, n (%)	10 (27.8)	2 (8.0)		0.099
Beta-blocker, n (%)	4 (11.1)	0 (0)		0.137

* Signiﬁcant differences.

† Of the 61 patients, the numbers evaluated for each test were as follows: MMSE 53; FAB 34; CDT 37; HDRS 61; OSIT-J 20.

‡ Details of medications are shown in online supplemental table e-3.

§ Details of cases with parkinsonism are shown in online supplemental table e-4.

ARWMC, age-related white matter change rating scale; CDT, the Clock Drawing Test; CIS, the cingulate island sign of brain perfusion SPECT; DAT-SPECT, dopamine transporter single-photon emission computed tomography; FAB, Frontal Assessment Battery; HDRS, Hamilton Depression Rating Scale; 123I-MIBG, 123I-metaiodobenzylguanidine myocardial scintigraphy; Late onset/early onset, onset of psychiatric disorder is after/less than 55 years of age; Midbrain atrophy, the area of the midbrain tegmentum area of <100.0 mm2 on mid-sagittal MRI; MMSE, The Mini-Mental State Examination; NaSSA, noradrenergic and specific serotonergic antidepressant; OSIT-J, the odour stick identification test for Japanese; SARI, serotonin antagonist and reuptake inhibitor; SNRI, selective serotonin–norepinephrine reuptake inhibitor; SSRI, selective serotonin reuptake inhibitor; Tetracyclic, tetracyclic antidepressants; Tricyclic, tricyclic antidepressants.

There were no differences in pre-existing medical conditions such as hypertension between the abnormal and the normal DAT-SPECT groups (online supplemental table e-5). For each psychiatric disease, 28/37 (75.7%) of MDD, 2/9 of schizophrenia, 3/7 of bipolar disorder, 1/4 of delusional disorder and 2/4 of anxiety disorder showed abnormality on DAT-SPECT (table 2).

**Table 2:** Characteristics of the patients for each mental disorder

	MDD (n=37)	Schizophrenia (n=9)	Bipolar disorder (n=7)	Delusional disorder (n=4)	Anxiety disorder (n=4)	P value
Sex, n (female/male)	27/10	7/2	5/2	3/1	1/3	0.359
Age in years, mean (SD)	72.1 (5.7)	67.2 (6.8)	74.0 (3.7)	75.3 (4.3)	77.3 (14.0)	0.057
Age at onset in years, mean (SD)	61.1 (11.7)	67.2 (6.8)	55.9 (16.8)	70.3 (4.0)	71.0 (17.0)	0.011*
Late onset/early onset (%)	29/8 (78.4)	3/6 (33.3)	3/4 (42.9)	4/0 (100.0)	3/1 (75.0)	0.027*
DAT-SPECT, abnormal/normal (%)	28/9 (75.7)	2/7 (22.2)	3/4 (42.9)	1/3 (25.0)	2/2 (50.0)	0.017*
^123^I-MIBG, abnormal/normal (%)	9/27 (25.0)	1/7 (12.5)	1/5 (16.7)	1/3 (25.0)	0/2 (0)	0.829
CIS, ± (%)	18/17 (51.4)	5/4 (55.6)	5/2 (71.4)	3/1 (75.0)	1/2 (33.3)	0.626
Midbrain atrophy, ± (%)	5/32 (13.5)	1/8 (11.1)	0/6 (0)	1/3 (25.0)	0/4 (0)	0.711

* Signiﬁcant differences.

CIS, the cingulate island sign of brain perfusion SPECT; DAT-SPECT, dopamine transporter single-photon emission computed tomography; 123I-MIBG, 123I-metaiodobenzylguanidine myocardial scintigraphy; Late onset/early onset, Onset of psychiatric disorder is after/less than 55 years of age; MDD, major depressive disorder; Midbrain atrophy, the area of the midbrain tegmentum area of <100.0 mm2 on mid-sagittal MRI.

Abnormal DAT-SPECT images were found in 29/42 (69.0%) of patients with late-onset psychiatric disorders (table 3). When limited to patients with late-onset MDD, 23/29 (79.3%) were abnormal on DAT-SPECT.

**Table 3:** Late-onset and early-onset geriatric psychiatric disorders

	Patients with late-onset psychiatric disorders (n=42)		Patients with early-onset psychiatric disorders (n=19)		*t*	P value
Sex, n (female/male)	29/13		14/5			0.771
Age in years, mean (SD)	74.2 (6.2)		67.6 (5.1)		−4.064	<0.001*
Age at onset in years, mean (SD)	67.2 (7.4)		43.1 (10.4)		−10.319	<0.001*
DAT-SPECT, abnormal/normal (%)	29/13 (69.0)		7/12 (36.8)			0.025*
^123^I-MIBG, abnormal/normal (%)	12/25 (32.4)		0/17 (0)			0.011*
	MDD (n=29)	Other than MDD (n=13)	MDD (n=8)	Other than MDD (n=11)		
DAT-SPECT, abnormal/normal (%)	23/6 (79.3)	6/7 (46.2)	5/3 (62.5)	2/9 (18.2)		
^123^I-MIBG, abnormal/normal (%)	9/18 (33.3)	3/7 (30.0)	0/7 (0)	0/10 (0)		

* Signiﬁcant differences.

DAT-SPECT, dopamine transporter single-photon emission computed tomography; 123I-MIBG, 123I-metaiodobenzylguanidine myocardial scintigraphy; MDD, major depressive disorder.

### ^123^I-MIBG myocardial scintigraphy

Of the 61 patients, 54 (88.5%) underwent ^123^I-MIBG scintigraphy (table 4). Of the 54 patients, 12 had abnormal ^123^I-MIBG scintigraphy, and all 12 who had abnormal ^123^I-MIBG scintigraphy also showed abnormal patterns on DAT-SPECT. There were no differences in scores of neuropsychological tests between the abnormal and normal ^123^I-MIBG scintigraphy groups. There were significantly more patients with late-onset psychiatric disorders in the abnormal ^123^I-MIBG scintigraphy group than in the normal group. There were significantly more patients with parkinsonism and RBD in the abnormal ^123^I-MIBG scintigraphy group compared with the normal group (table 4). There were no differences in other clinical findings such as psychiatric symptoms, autonomic symptoms and olfactory disturbance between the abnormal and normal ^123^I-MIBG scintigraphy groups (online supplemental table e-6). Abnormal ^123^I-MIBG scintigraphy was found in 12/37 (32.4%) of patients with late-onset psychiatric disorders, while none of the early-onset cases had it. There was no significant difference in the percentage of patients between the normal and abnormal ^123^I-MIBG scintigraphy groups who were taking medications that may reduce ^123^I-MIBG cardiac uptake such as SNRIs or tricyclic antidepressants (table 4), and who have a medical history that may reduce ^123^I-MIBG uptake such as heart disease or diabetes (online supplemental table e-8).

**Table 4:** Characteristics of the patients who underwent ^123^I-MIBG scintigraphy

	Abnormal ^123^I-MIBG (n=12)	Normal ^123^I-MIBG (n=42)	*t*	P value
Sex, n (female/male)	9/3	30/12		1.000
Age in years, mean (SD)	73.7 (4.3)	71.1 (6.6)	1.285	0.204
Age at onset in years, mean (SD)	67.1 (8.8)	57.9 (14.3)		0.041*
Late onset/early onset (%)	12/0 (100)	25/17 (59.5)		0.008*
Education in years, mean (SD)	12.7 (2.5)	12.9 (2.7)	−0.148	0.883
DAT-SPECT, abnormal/normal (%)	12/0 (100%)	20/22 (47.6)		0.001*
CIS, ± (%)	5/6 (45.5)	26/16 (61.9)		0.493
ARWMC score, more than 5/less than 5 (%)	7/5 (58.3)	15/27 (35.7)		0.194
ARWMC score of basal ganglia, 1 and above / 0 (%)	4/8 (33.3)	7/35 (16.7)		0.237
Midbrain atrophy, ± (%)	4/7 (36.4)	3/39 (7.1)		0.011*
Neuropsychological tests†				
HDRS, mean (SD)	19.6±7.7	18.0±9.4	0.547	0.587
MMSE, mean (SD)	25.8±3.8	26.3±3.0	−0.440	0.662
FAB, mean (SD)	12.6±1.5	14.1±2.8	−1.488	0.147
CDT, mean (SD)	8.9±1.4	8.9±1.5	0.076	0.940
OSIT-J, mean (SD)	4.3±3.2	5.1±3.1	−0.400	0.694
Clinical symptoms				
Depression, n (%)	10 (83.3)	32 (76.2)		0.714
Anxiety, n (%)	7 (58.3)	30 (71.4)		0.389
Delusion, n (%)	6 (50.0)	22 (52.4)		0.884
Fluctuations, n (%)	3 (25.0)	4 (9.5)		0.175
Parkinsonism, n (%)	10 (83.3)	7 (16.7)		<0.001*
Visual hallucination, n (%)	2 (16.7)	1 (2.4)		0.121
REM sleep behaviour disorder, n (%)	2 (16.7)	0 (0)		0.046*
Medications‡				
SSRI, n (%)	5 (41.7)	11 (26.2)		0.309
SNRI, n (%)	3 (25.0)	3 (7.1)		0.116
NaSSA, n (%)	0 (0)	13 (31.0)		0.050
Tricyclic, n (%)	1 (8.3)	0 (0)		0.222
Tetracyclic, n (%)	0 (0)	0 (0)		–
SARI, n (%)	4 (33.3)	5 (11.9)		0.098
Benzodiazepine, n (%)	6 (50.0)	28 (66.7)		0.292
Mood stabiliser, n (%)	2 (16.7)	4 (9.5)		0.605
Antipsychotic drug, n (%)	5 (41.7)	23 (54.8)		0.520
Calcium-blocker, n (%)	3 (25.0)	6 (14.3)		0.380
Beta-blocker, n (%)	1 (8.3)	3 (7.1)		1.000

* Signiﬁcant differences.

† Of the 54 patients, the numbers evaluated for each test were as follows: HDRS 54; MMSE 47; FAB 32; CDT 35; OSIT-J 20.

‡ Details of medications are shown in online supplemental table e-7.

ARWMC, the Age-Related White Matter Change Rating Scale; CDT, the Clock Drawing Test; CIS, the cingulate island sign of brain perfusion SPECT; DAT-SPECT, dopamine transporter single-photon emission computed tomography; FAB, Frontal Assessment Battery; HDRS, Hamilton Depression Rating Scale; 123I-MIBG, 123I-metaiodobenzylguanidine myocardial scintigraphy; Late onset/early onset, Onset of psychiatric disorder is after/less than 55 years of age; Midbrain atrophy, the area of the midbrain tegmentum area of <100.0 mm2 on mid-sagittal MRI; MMSE, The Mini-Mental State Examination; NaSSA, noradrenergic and specific serotonergic antidepressant; OSIT-J, the odour stick identification test for Japanese; SARI, serotonin antagonist and reuptake inhibitor; SNRI, selective serotonin–norepinephrine reuptake inhibitor; SSRI, selective serotonin reuptake inhibitor; Tetracyclic, tetracyclic antidepressants; Tricyclic, tricyclic antidepressants.

### Vascular lesions

Vascular lesions in the white matter of each brain region were evaluated for all 61 patients. Patients with a total ARWMC score of 5 or higher and patients with white matter changes in the basal ganglia were significantly more common in the abnormal DAT-SPECT group than in the normal group (table 1). Furthermore, all patients who had white matter changes in the basal ganglia showed abnormal scores on DAT-SPECT.

### Measurements of midbrain area

60 of 61 patients (98.3%) who underwent DAT-SPECT had also undergone MRI and had midsagittal T1-weighted images. 7 of the 60 patients (11.7%) showed midbrain atrophy (midbrain tegmentum area of <100.0 mm^2^). The percentage of patients with midbrain atrophy did not differ between the abnormal and normal DAT-SPECT groups. The group with abnormal ^123^I-MIBG scintigraphy clearly included more patients with midbrain atrophy than the group with normal ^123^I-MIBG scintigraphy (table 4).

### Cingulate island sign

58 of 61 patients (95.1%) who had undergone DAT-SPECT and53 of the 54 patients (98.1%) who underwent ^123^I-MIBG scintigraphy also underwent brain perfusion scintigraphy. There was no difference in the proportion of cases showing CIS between the abnormal and normal DAT-SPECT groups (table 1), nor between the normal and abnormal ^123^I-MIBG scintigraphy groups (table 4).

## Discussion

### A high percentage of patients with geriatric psychiatric disorders showed abnormalities on DAT-SPECT

It has long been assumed that some patients with geriatric psychiatric disorders present with psychiatric symptoms as symptoms of the prodromal phase of neurodegenerative diseases.^29 30^ The reported rate of DLB in the population of all dementias is about 7.5%.^31^ The prevalence of other neurodegenerative diseases that present abnormalities on DAT-SPECT such as PSP and CBD is very small. Therefore, our result of 36/61 (59%) DAT abnormality in inpatients with primary geriatric psychiatric disorders is remarkably higher than previous reports. The possible reasons are as follows: first, there could be a large number of patients with geriatric psychiatric disorders who fall into the precursor phase of neurodegenerative diseases characterised by nigrostriatal dopaminergic deficit. Second, factors other than neurodegenerative diseases may affect DAT-SPECT. Cerebrovascular disease could have affected the DAT-SPECT results. In fact, there were significantly more cases with a high ARWMC (≥5) score and cases with ARWMC in the basal ganglia in the abnormal DAT-SPECT group than in the normal group. A study of patients with vascular parkinsonism has reported that even in the absence of ischaemic lesions on the striatum and the presence of only subcortical white matter hyperintensities, quantitative assessment of DAT binding on ^18^F-FP-CIT positron emission tomography (PET) showed that it was decreased in most striatal regions.^32^ In addition, because the participants were under psychiatric treatment and the DAT-SPECT was performed while they were receiving psychotropic drugs, it is possible that the medications may have affected the DAT-SPECT results.

### Patterns of ^123^I-MIBG and DAT-SPECT findings in patients with geriatric psychiatric disorders

**Figure 2:**
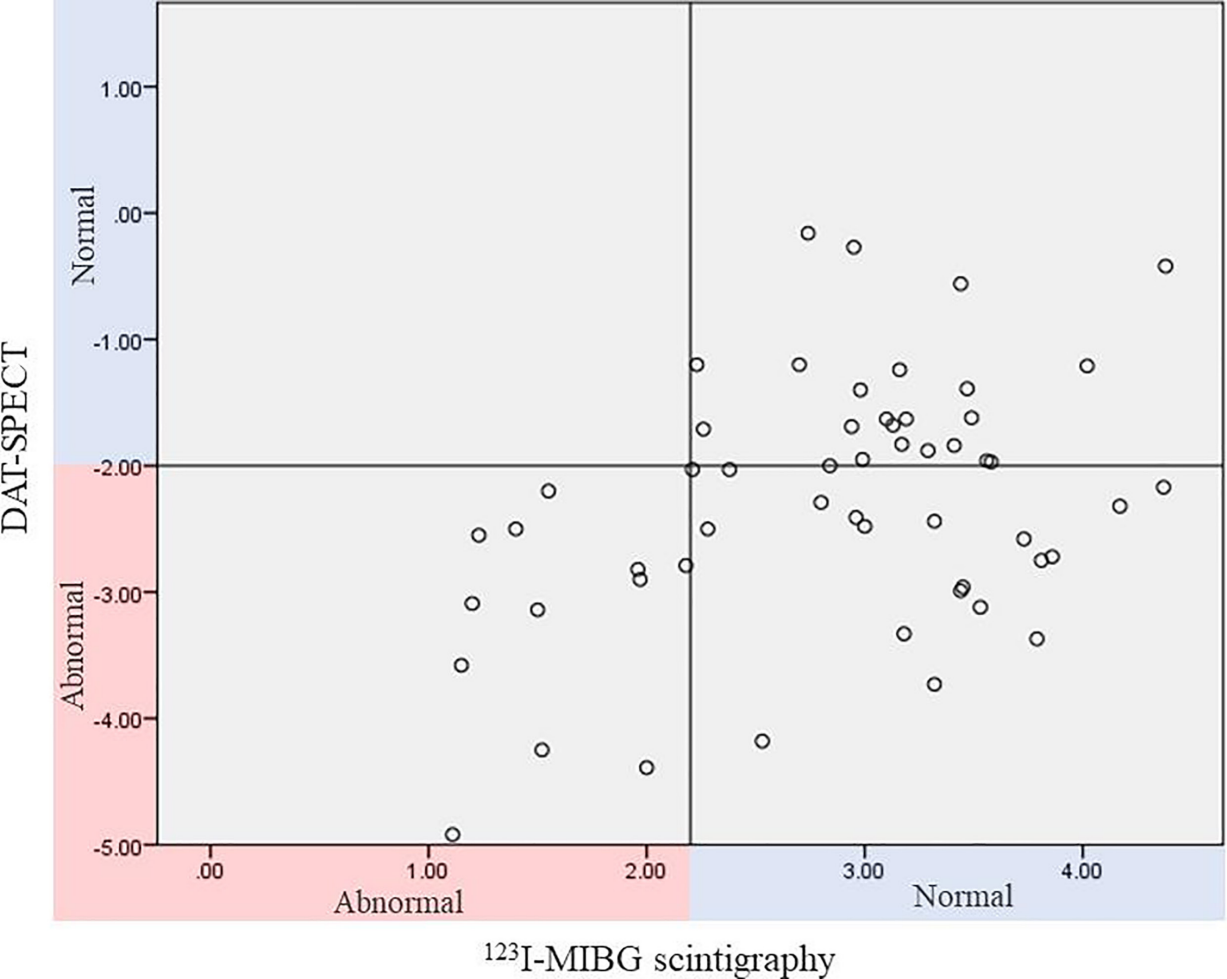
The plot on the vertical axis (DAT-SPECT) shows the SD value for each patient. This study defined the SD value of lower side specific binding ratio (SBR) as less than −2 SD of the mean value calculated from the value of the control group as abnormal. The plot on the horizontal axis (^123^I-MIBG scintigraphy) shows the [heart-to-mediastinum (H/M) ratio of ^123^I-MIBG scintigraphy in the late image] for each patient. This study defines a group with a H/M ratio of less than 2.2 in the late image as abnormal. DAT-SPECT, dopamine transporter single-photon emission computed tomography; ^123^I-MIBG scintigraphy, ^123^I-metaiodobenzylguanidine myocardial scintigraphy.

Among the patients with geriatric psychiatric disorders included in this study, there were patients with abnormalities on both ^123^I-MIBG scintigraphy and DAT-SPECT, and patients with normal ^123^I-MIBG scintigraphy and abnormal DAT-SPECT, but none of the patients had abnormal ^123^I-MIBG scintigraphy and normal DAT-SPECT (figure 2). It is surprising to find such a consistent and pronounced trend in a study with sample sizes of more than 60.

There are four possible reasons why many patients had normal ^123^I-MIBG scintigraphy and only DAT-SPECT was abnormal. First, the study may have included patients with a number of neurodegenerative diseases that are abnormal on DAT-SPECT but not on ^123^I-MIBG scintigraphy (MSA for synucleinopathy, PSP and CBD for tauopathy). Second, it is possible that many cases of LBD in which ^123^I-MIBG scintigraphy has not yet shown a decrease were included in the study. For example, DLB is the endpoint of one form of LBD, and has a higher percentage of abnormalities on both DAT-SPECT and ^123^I-MIBG scintigraphy (sensitivity of 77.7% for DAT-SPECT and 77–80% for ^123^I-MIBG scintigraphy).^12 33 34^ On the other hand, in idiopathic RBD, which is considered one of the early clinical phenotypes of LBD,^35^ a low percentage of abnormalities on DAT-SPECT (44%) and a high percentage of abnormalities on ^123^I-MIBG scintigraphy (91%) have been reported.^36 37^ What kind of biomarker abnormality patterns are seen in psychiatric-onset prodromal DLB has not been clarified. Assuming that the patients who were the subjects of this study include a large number with psychiatric-onset prodromal DLB, cases that are abnormal on DAT-SPECT and normal on ^123^I-MIBG scintigraphy in the early stage and show abnormal results on ^123^I-MIBG scintigraphy become increasingly apparent in later stages may be the majority in psychiatric-onset prodromal DLB. Third, cerebrovascular disease does not directly affect ^123^I-MIBG but does affect DAT-SPECT, and this possibility must be considered. Fourth, it is possible that the medications that the participants were taking while undergoing testing may have affected the outcomes of both the DAT-SPECT and the ^123^I-MIBG scintigraphy test results. Since this is a cross-sectional study, no definitive conclusions can be drawn on the question of the extent to which the study population includes cases in the prodromal phase of neurodegenerative disease. Future longitudinal studies are needed to better clarify this question.

### Patients with late-onset psychiatric disorders and MDD present more frequently with abnormalities on DAT-SPECT

A significantly higher proportion of patients in the late-onset psychiatric disorders group showed abnormalities on DAT-SPECT, and a higher proportion of patients showed abnormalities on ^123^I-MIBG scintigraphy compared with the early-onset psychiatric disorders group. These results suggest that the physiologic mechanisms underlying psychiatric disorders differ between early-onset psychiatric disorders and late-onset psychiatric disorders groups (especially MDD) and provide a basis for the opinion that there is a need to differentiate treatment approaches for these two groups.

### Effects of medications on DAT-SPECT and ^123^I-MIBG

This study was an observational study, and the treatment of the participants was a priority. Therefore, some patients were taking medications that had the potential to affect the results of examinations (online supplemental tables e-3 and e-7). Previous studies have clearly shown that certain psychotropic medications, such as psychostimulants and bupropion, can affect DAT-SPECT results.^38^ On the other hand, the effects of other antidepressants such as SSRIs and SNRIs on DAT-SPECT results have been inconsistent in the results across studies and remain unclear.^38^ In this study, SSRI users and SNRI users were significantly more likely to be in the abnormal DAT-SPECT group than in the normal group. The large number of anti-depressant users in the abnormal DAT-SPECT group was probably a result of the fact that the abnormal DAT-SPECT group contained many MDD patients. However, several studies have indicated that these medications may affect DAT-SPECT results, and the possibility that medications may have affected the results of DAT-SPECT cannot be ruled out. It is recommended that withholding of tricyclic antidepressants, SNRIs, one of the beta blockers (labetalol), and reserpine be considered prior to ^123^I-MIBG scintigraphy.^39^ In addition, although prior drug withdrawal is not recommended, pharmacological mechanisms and several research reports suggest that calcium blockers and antipsychotics may affect ^123^I-MIBG scintigraphy results.^39^ In this study, some patients (7/54) underwent ^123^I-MIBG scintigraphy while taking SNRIs or TCAs, which have been shown to produce artificially low cardiac uptake of ^123^I-MIBG in scintigraphy. Although there was no significant difference in the percentage of patients between the normal and abnormal ^123^I-MIBG scintigraphy groups who were taking these medications, it is possible that the abnormal ^123^I-MIBG scintigraphy patients in this study included a false-positive case.

### Limitations

First, DAT-SPECT and ^123^I-MIBG scintigraphy abnormalities do not directly indicate the presence of a specific brain pathology. Second, this is a cross-sectional study. Third, in routine medical practice, a combination of visual assessment and semi-quantitative assessment is used to evaluate DAT-SPECT, whereas in this study only semi-quantitative evaluation was used. A visual assessment of DAT-SPECT is important to determine whether it is LBD or not, and the use of a visual assessment method is an issue for consideration in future studies. Fourth, the effects of medications on DAT-SPECT and ^123^I-MIBG results cannot be ruled out. Fifth, the participants of this study were inpatients of a single-site tertiary care institution. Further studies are needed to confirm whether the results can be generalised to cases involving outpatients.

## Conclusion

Patients with geriatric psychiatric disorders include many cases showing abnormalities on DAT-SPECT. DAT-SPECT abnormalities were more frequent in patients with late-onset psychiatric disorders and MDD, especially late-onset MDD. It suggests that they have a high risk of developing neurodegenerative diseases characterised by a dopaminergic deficit. There were many cases of geriatric psychiatric disorders with abnormalities on DAT-SPECT, while there was no case with normal DAT-SPECT and abnormal ^123^I-MIBG scintigraphy only. It is possible that neurodegenerative diseases whose phenotype in the early stages of the disease is a psychiatric disorder tend to show abnormalities on DAT-SPECT first rather than on ^123^I-MIBG scintigraphy.

## Supplementary material

10.1136/bmjment-2024-301042 online supplemental file 1

## Data Availability

Data are available upon reasonable request.
