# Supplementary material for: Potential dopaminergic deficit in patients with geriatric psychiatric disorders as revealed by DAT-SPECT: a cross-sectional study
Source: BMJ Ment Health. 2024 Jul 30;27(1):e301042. doi: 10.1136/bmjment-2024-301042 (PMC11293386; doi:10.1136/bmjment-2024-301042)
Supplement: online supplemental file 1 [file bmjment-27-1-s001.docx]

**Supplemental table e1 Potential participants and subject of this study**

|  | Potential participants (n=265) | Excluded patients (n=204) | Included patients (n=61) |
| --- | --- | --- | --- |
| Sex, n (female/male) | 157/108 | 114/90 | 42/19 |
| Age in years, mean (SD) | 70.9 (7.2) | 70.6 (7.3) | 72.1 (6.8) |
| F0*: Organic, including symptomatic, mental disorders, n (%) | 51 (19.2) | 51 (25.0) | 0 (0) |
| F2*: Schizophrenia, schizotypal and delusional disorders, n (%) | 57 (21.5) | 44 (21.6) | 13 (21.3) |
| F3*: Mood (affective) disorders, n (%) | 111 (41.8) | 67 (32.8) | 44 (72.1) |
| F4*: Neurotic, stress-related and somatoform disorders, n (%) | 20 (7.5) | 16 (6.0) | 4 (6.6) |
| Others, n (%) | 26 (9.8) | 26 (12.7) | 0 (0) |
| *ICD-10 diagnostic criteria were used to classify the diagnosis. World Health Organization. The ICD-10 Classification of Mental and Behavioral Disorders: Diagnostic Criteria for Research. Geneva: World Health Organization 1993. | | | |

**Supplemental table e2 Clinical symptoms of the patients who underwent DAT-SPECT**

|  | Abnormal DAT-SPECT | Normal DAT-SPECT | p |
| --- | --- | --- | --- |
| Depression, n (%) | 31(86.1) | 15(60.0) | 0.020* |
| Suicide attempt, n (%) | 11(30.6) | 5(20.0) | 0.300 |
| Suicidal ideation, n (%) | 26(72.2) | 10(40.0) | 0.232 |
| Anxiety, n (%) | 22(61.1) | 19(76.0) | 0.223 |
| Delusion, n (%) | 15(41.7) | 16(64.0) | 0.086 |
| Fluctuations, n (%) | 5(13.9) | 5(20.0) | 0.727 |
| Parkinsonism, n (%) | 13(36.1) | 6(24.0) | 0.315 |
| Visual hallucination, n (%) | 2(5.6) | 2(8.0) | 1.000 |
| Auditory hallucination, n (%) | 4(11.1) | 4(16.0) | 0.706 |
| REM sleep behavior disorder, n (%) | 2(5.6) | 0(0) | 0.508 |
| Olfactory disturbance, n (%) | 3(8.3) | 2(8.0) | 1.000 |
| Salivary-secretion abnormality, n (%) | 2(5.6) | 0(0) | 0.508 |
| Orthostatic hypotension, n (%) | 5(13.9) | 0(0) | 0.072 |
| Loss-of-consciousness, n (%) | 0(0) | 1(4.0) | 0.410 |
| Constipation, n (%) | 23(63.9) | 13(52.0) | 0.353 |
| Dysuria, n (%) | 4(11.1) | 4(16.0) | 0.706 |
| ^*^Signiﬁcant differences. DAT-SPECT, ^123^I-FP-CIT dopamine transporter single photon emission computed tomography | | | |

**Supplemental table e3 Neuroleptics and white matter changes in patients with and without parkinsonism**

|  | Patients with parkinsonism (n=19) | Patients without parkinsonism (n=42) | p |
| --- | --- | --- | --- |
| Neuroleptics (Antipsychotic drug or amoxapine) ^*^ | 9 (47.4) | 23 (54.8) | 0.592 |
| ARWMC score, more than 5/less than 5 (%) | 9/10 (47.4) | 18/24 (42.9) | 0.743 |
| ARWMC score of basal ganglia, 1 and above/0 (%) | 5/14 (26.3) | 8/34 (19.0) | 0.521 |
| ARWMC, the Age-Related White Matter Change Rating Scale. *We have not examined all medications that can produce parkinsonism such as the dopamine receptor blockers used in gastrointestinal therapy. The relationship between the timing of drug initiation and the onset of parkinsonism is unknown. | | | |
|  |  |  |  |
|  |  |  |  |

**Supplemental table e4 Medication details and DAT-SPECT**

|  | Abnormal DAT-SPECT (n=36) | Normal DAT-SPECT (n=25) | p |
| --- | --- | --- | --- |
| SSRI, n (%) | 16(44.4) | 3(12.0) | 0.007* |
| Sertraline | 4 | 1 |  |
| Escitalopram | 8 | 1 |  |
| Paroxetine | 2 | 1 |  |
| Fluvoxamine | 1 | 0 |  |
| Vortioxetine | 1 | 0 |  |
| SNRI, n (%) | 7(19.4) | 0(0) | 0.019* |
| Duloxetine | 5 | 0 |  |
| Venlafaxine | 2 | 0 |  |
| NaSSA, n (%) | 8(22.2) | 5(20.0) | 1.000 |
| Mirtazapine | 8 | 5 |  |
| Tricyclic, n (%) | 1(2.8) | 0(0) | 0.500 |
| Amoxapine | 1 | 0 |  |
| Tetracyclic, n (%) | 0(0) | 0(0) | - |
| SARI, n (%) | 8(22.2) | 3(12.0) | 0.500 |
| Trazodone | 8 | 3 |  |
| Benzodiazepine, n (%) | 18(50.0) | 20(80.0) | 0.017* |
| Mood stabilizer, n (%) | 6(16.7) | 4(16.0) | 1.000 |
| Carbamazepine | 2 | 0 |  |
| Lamotrigine | 2 | 1 |  |
| Lithium carbonate | 1 | 2 |  |
| Valproic acid | 1 | 1 |  |
| Antipsychotic drug, n (%) | 17(47.2) | 14(56.0)† | 0.500 |
| Quetiapine | 10 | 6 |  |
| Aripiprazole | 3 | 0 |  |
| Brexpiprazole | 0 | 2 |  |
| Olanzapine | 0 | 2 |  |
| Perospirone | 0 | 2 |  |
| Risperidone | 2 | 4 |  |
| Levomepromazine | 1 | 1 |  |
| Ziprasidone | 1 | 0 |  |
| Calcium-blocker, n (%) | 10(27.8) | 2(8.0) | 0.099 |
| Amlodipine | 9 | 2 |  |
| Azelnidipine | 1 | 0 |  |
| Beta-blocker, n (%) | 4(11.1) | 0(0) | 0.137 |
| Carvedilol | 2 | 0 |  |
| Bisoprolol | 1 | 0 |  |
| *Signiﬁcant differences. DAT-SPECT, ^123^I-FP-CIT dopamine transporter single photon emission computed tomography. †Three participants were on multiple concomitant antipsychotic medications. | | | |

**Supplemental table e5 Pre-existing medical conditions of the patients who underwent DAT-SPECT**

|  | Abnormal DAT-SPECT | Normal DAT-SPECT | p |
| --- | --- | --- | --- |
| Stroke, n (%) | 2（5.6） | 1（4.0） | 1.000 |
| Heart disease, n (%) | 0（0） | 0（0） | - |
| Hypertension, n (%) | 16(44.4) | 6(24.0) | 0.102 |
| Diabetes mellitus, n (%) | 4(11.1) | 4(16.0) | 0.706 |
| *Signiﬁcant differences. DAT-SPECT, ^123^I-FP-CIT dopamine transporter single photon emission computed tomography | | | |

**Supplemental table e6 Clinical symptoms of the patients who underwent ^123^I-MIBG scintigraphy**

|  | Abnormal ^123^I-MIBG | Normal ^123^I-MIBG | p |
| --- | --- | --- | --- |
| Depression, n (%) | 10(83.3) | 32(76.2) | 0.714 |
| Suicide attempt, n (%) | 2(16.7) | 13(31.0) | 0.474 |
| Suicidal ideation, n (%) | 3(25.0) | 24(57.1) | 0.099 |
| Anxiety, n (%) | 7(58.3) | 30(71.4) | 0.389 |
| Delusion, n (%) | 6(50.0) | 22(52.4) | 0.884 |
| Fluctuations, n (%) | 3(25.0) | 4(9.5) | 0.175 |
| Parkinsonism, n (%) | 10(83.3) | 7(16.7) | <0.001* |
| Visual hallucination, n (%) | 2(16.7) | 1(2.4) | 0.121 |
| Auditory hallucination, n (%) | 3(25.0) | 5(11.9) | 0.356 |
| REM sleep behavior disorder, n (%) | 2(16.7) | 0(0) | 0.046* |
| Olfactory disturbance, n (%) | 2(16.7) | 2(4.8) | 0.210 |
| Salivary-secretion abnormality, n (%) | 1(8.3) | 1(2.4) | 0.398 |
| Orthostatic hypotension, n (%) | 3(25.0) | 2(4.8) | 0.067 |
| Loss-of-consciousness, n (%) | 0(0) | 1(2.4) | 1.000 |
| Constipation, n (%) | 10(83.3) | 22(52.4) | 0.094 |
| Dysuria, n (%) | 2(16.7) | 5(11.9) | 0.645 |
| *Signiﬁcant differences. ^123^I-MIBG, ^123^I-metaiodobenzylguanidine myocardial scintigraphy | | | |

**Supplemental table e7 Medication details and ^123^I-MIBG scintigraphy**

|  | Abnormal ^123^I-MIBG (n=12) | Normal ^123^I-MIBG (n=42) | p |
| --- | --- | --- | --- |
| SSRI, n (%) | 5(41.7) | 11(26.2) | 0.309 |
| Sertraline | 2 | 1 |  |
| Escitalopram | 1 | 7 |  |
| Paroxetine | 1 | 2 |  |
| Fluvoxamine | 0 | 1 |  |
| Vortioxetine | 1 | 0 |  |
| SNRI, n (%) | 3(25.0) | 3(7.1) | 0.116 |
| Duloxetine | 2 | 3 |  |
| Venlafaxine | 1 | 0 |  |
| NaSSA, n (%) | 0(0) | 13(31.0) | 0.050* |
| Mirtazapine | 0 | 13 |  |
| Tricyclic, n (%) | 1(8.3) | 0(0) | 0.222 |
| Amoxapine | 1 | 0 |  |
| Tetracyclic, n (%) | 0(0) | 0(0) | - |
| SARI, n (%) | 4(33.3) | 5(11.9) | 0.098 |
| Trazodone | 4 | 5 |  |
| Benzodiazepine, n (%) | 6(50.0) | 28(66.7) | 0.292 |
| Mood stabilizer, n (%) | 2(16.7) | 4(9.5) | 0.605 |
| Carbamazepine | 0 | 0 |  |
| Lamotrigine | 1 | 2 |  |
| Lithium carbonate | 0 | 2 |  |
| Valproic acid | 1 | 0 |  |
| Antipsychotic drug, n (%) | 5(41.7) | 23(54.8)† | 0.520 |
| Quetiapine | 3 | 11 |  |
| Aripiprazole | 1 | 2 |  |
| Brexpiprazole | 0 | 2 |  |
| Olanzapine | 0 | 2 |  |
| Perospirone | 0 | 2 |  |
| Risperidone | 1 | 4 |  |
| Levomepromazine | 0 | 2 |  |
| Ziprasidone | 0 | 1 |  |
| Calcium-blocker, n (%) | 3(25.0) | 6(14.3) | 0.380 |
| Amlodipine | 3 | 6 |  |
| Azelnidipine | 0 | 0 |  |
| Beta-blocker, n (%) | 1(8.3) | 3(7.1) | 1.000 |
| Carvedilol | 0 | 3 |  |
| Bisoprolol | 1 | 0 |  |
| *Signiﬁcant differences. ^123^I-MIBG, ^123^I-metaiodobenzylguanidine myocardial scintigraphy. †Three participants were on multiple concomitant antipsychotic medications. | | | |

**Supplemental table e8 Pre-existing medical conditions of the patients who underwent ^123^I-MIBG scintigraphy**

|  | Abnormal ^123^I-MIBG | Normal ^123^I-MIBG | p |
| --- | --- | --- | --- |
| Stroke, n (%) | 1(8.3) | 2(4.8) | 0.537 |
| Heart disease, n (%) | 0(0) | 0(0) | - |
| Hypertension, n (%) | 6(50.0) | 13(31.0) | 0.223 |
| Diabetes mellitus, n (%) | 1(8.3) | 5(11.9) | 1.000 |
| *Signiﬁcant differences. ^123^I-MIBG, ^123^I-metaiodobenzylguanidine myocardial scintigraphy | | | |
